# Cross-linguistic transfer in bilingual children’s phonological and morphological awareness skills: a longitudinal perspective

**DOI:** 10.1017/s1366728924000439

**Published:** 2024-09-26

**Authors:** Kehui Zhang, Xin Sun, Zahira Flores-Gaona, Chi-Lin Yu, Rachel L. Eggleston, Nia Nickerson, Valeria C. Caruso, Twila Tardif, Ioulia Kovelman

**Affiliations:** 1 Division of Arts and Sciences, New York University (NYU) Shanghai, Shanghai, China;; 2 Department of Psychology, University of British Columbia, Vancouver, BC, Canada;; 3 Department of Psychology, University of Michigan, Ann Arbor, MI, USA; 4 Department of Psychiatry, University of Michigan, Ann Arbor, MI, USA

**Keywords:** bilingualism, phonological awareness, morphological awareness, word reading, longitudinal

## Abstract

Cross-linguistic interactions are the hallmark of bilingual development. Theoretical perspectives highlight the key role of *cross-linguistic distances* and *language structure* in literacy development. Despite the strong theoretical assumptions, the impact of such bilingualism factors in heritage-language speakers remains elusive given high variability in children’s heritage-language experiences. A longitudinal inquiry of heritage-language learners of structurally distinct languages – Spanish–English and Chinese–English bilinguals (*N* = 181, *M*_age_ = 7.57, measured 1.5 years apart) aimed to fill this gap. Spanish–English bilinguals showed stronger associations between morphological awareness skills across their two languages, across time, likely reflecting cross-linguistic similarities in vocabulary and lexical morphology between Spanish and English. Chinese–English bilinguals, however, showed stronger associations between morphological and word reading skills in English, likely reflecting the critical role of morphology in spoken and written Chinese word structure. The findings inform theories of literacy by uncovering the mechanisms by which bilingualism factors influence child literacy development.

## 1. Introduction

Across languages, children’s learning to read builds upon phonological and morphological literacy skills, that is, the ability to recognize phonemes (or units of sounds in speech) and morphemes (or units of meaning in speech). However, there is substantial cross-linguistic variation in how phonemes and morphemes map onto print across alphabet-based orthographies, such as English and Spanish, and character-based orthographies, such as Chinese. In Spanish and English, individual phonemes typically map onto letters, whereas in Chinese, individual morphemes map onto characters. Such cross-linguistic variation is known to influence the developmental trajectories for learning to read in monolingual children. Yet, many of the world’s young children learn to read in more than one language, including Spanish–English and Chinese–English bilinguals growing up in the United States. This study leverages cross-linguistic variation between Spanish and Chinese to uncover universal and language-specific influences of bilingualism on children’s developmental trajectories for learning to read. We ask: *How do cross-linguistic differences between a child two languages influence a bilingual child*’*s word-reading development over time?*

### 1.1. Word reading in monolinguals across languages

English and Spanish both employ alphabetic script systems, but they differ in the predictability of sound-to-print associations. English typically demonstrates less straightforward correlations between sounds and letters, making advancement in complex morpho-phonological awareness skills crucial for word reading (Ziegler & Goswami, 2005). For instance, knowing stable morpho-phonological associations for units such as “magic” are essential for reading and spelling words such as “magician.” In contrast, Spanish is known for its highly predictable sound-to-print associations, making it one of the most transparent alphabetic languages. For example, in Spanish, the word “gato” (cat) maps directly and consistently onto the letters g-a-t-o. This means that phonological skills, such as the ability to segment and manipulate sounds, play a significant role in Spanish literacy in a way that is similar to but more straightforward than in English.

Differently from Spanish and English, Chinese employs a morpho-syllabic script system in which lexical morphemes map onto characters. This script system interacts with Chinese spoken word structure, which is predominantly that of lexical compounds. For instance, the word 飞机 (fei1 ji1, meaning “airplane”) comprises the morphemes 飞 (fei1, “to fly”) and 机 (ji1, “machine”) (Kuo & Anderson, 2006). Note how each morpheme of the compound word maps onto an individual character. Moreover, a key feature of Chinese characters is the presence of radicals that provide phonetic and semantic cues. Although most characters (~80–90%) contain both semantic and phonetic radicals (Ho & Bryant, 1997), the predictive accuracy of semantic radicals in determining character meaning is substantially higher than that of phonetics (Chung & Leung, 2008; Feldman & Siok, 1999; Shu et al., 2003). In sum, the spoken and written structures of Chinese words interact to yield a complex orthographic system in which morphology plays a key role.

#### 1.1.1. Developmental changes in learning to read

Models of word reading, such as the lexical quality hypothesis (Perfetti & Hart, 2002; Verhoeven & Perfetti, 2021), suggest that reading a word involves the interaction of three representations: phonological, orthographical and morpho-syntactic, yielding meaning-to-sound, sound-to-print and meaning-to-print associations (often depicted as a “triangle” of interdependencies). This framework is based on several core assumptions. First, that the three core elements (phonological, morphological and orthographic) interact, reciprocally strengthening each other and their associations. Second, there are stronger and weaker players in the triangle (e.g., phonological skills being stronger in English and Spanish, morphological skills being more vital in Chinese). Finally, the relative strength or contribution of a given literacy skill can change over time. For instance, in the case of alphabetic learners, phonological skills are foundational, often propelling the development of other literacy skills, whereas morphological skills grow in relevance over time (Deacon & Kirby, 2004).

#### 1.1.2. Concurrent and longitudinal literacy in English

*Phonological awareness* is the stepping stone to learning to read across languages. Several decades of research has demonstrated the critical role of phonological awareness in early reading gains in English, often exceeding the role of morphological awareness in early grades (Adams, 1994; Goswami & Bryant, 2016). *Morphological awareness*’*s* contribution to word reading in languages such as English usually starts small, smaller than that of phonological awareness, but it grows steadily over time (Bowers et al., 2010; Carlisle, 2003; Deacon & Kirby, 2004; Kirby et al., 2012; Levesque & Deacon, 2022; Marks et al., 2023). Importantly, both phonological and morphological awareness appear to make a longitudinal contribution to children’s word reading across grades (phonology: Deacon & Kirby, 2004; Hogan et al., 2005; Landerl et al., 2019; morphology: Bowers et al., 2010; Deacon et al., 2013; Kirby et al., 2012; Law & Ghesquière, 2021; Levesque & Deacon, 2022).

#### 1.1.3. Concurrent and longitudinal literacy in Spanish

Although some researchers have posited that *phonological awareness* might be less relevant to learning to read in Spanish due to high phonological transparency and a relatively simple syllable structure, a recent meta-analysis review paper by Míguez-Álvarez et al. (2022) found that the concurrent contributions of phonological awareness to children’s emergent word-reading skills in Spanish were comparable to those previously reported in similar meta-analysis of English word reading (Swanson et al., 2003).

The *morphological structure* of words in Spanish is similar to English and other Indo-European languages, which includes a rich array of derivational and compound word structures. The derivational morphology is generally more productive in Romance languages, such as Spanish, French, and Italian, than English (cf. Duncan et al., 2009). Notably, Spanish and English have similar levels of morpheme-to-print predictability, especially for affixes, offering a similar level of consistency to support visual word recognition in the two languages. It is, therefore, possible that readers of the two languages are guided by similar mechanisms to use morphological knowledge to learn to read. Importantly, research generally suggests that young readers of Romance languages such as Spanish and Italian use morphological information above and beyond the relatively transparent phonological information (Burani et al., 2002; D’Alessio et al., 2019; Lázaro et al., 2021). Unfortunately, the longitudinal relationships between phonological or morphological awareness and children’s word reading in Spanish are less explored (Kim & Pallante, 2012) – an evidence gap our work aims to fill.

#### 1.1.4. Concurrent and longitudinal literacy in Chinese

*Phonological awareness* plays a critical role in Chinese literacy acquisition, with empirical evidence underscoring the importance of syllable awareness in character reading (Pan et al., 2011, 2016; Tong et al., 2011). Phonemic awareness, as indicated by phoneme deletion tasks, predicts character reading success and identifies dyslexia (Pan & Shu, 2014; Xue et al., 2013). Notably, an 8-year longitudinal study in mainland China found syllable awareness in early childhood (ages 4–6) to be a unique predictor of later character reading and morphological awareness (Pan et al., 2016). However, in contrast to English, the significance of phoneme awareness in Chinese is comparatively less, as revealed in a study contrasting bilingual children’s word-reading development in Chinese and English (McBride et al., 2008).

*Morphological awareness*, essential for Chinese character reading, both concurrently and longitudinally influences literacy development (McBride-Chang et al., 2003; Pan et al., 2016; Shu et al., 2006; Tong et al., 2017; Yeung et al., 2013). Yeung et al.’s (2013) 3-year study highlighted morphological construction as the strongest predictor of word-reading development. Furthermore, Tong et al. (2017) found morphological awareness more closely associated with reading and spelling difficulties than phonological awareness, differing from patterns observed in alphabetic languages.

Similar to alphabetic languages, there is evidence of a reciprocal longitudinal relationship between phonological and morphological awareness in Chinese literacy development. Preliterate syllable awareness has been shown to predict morphological awareness years later, underscoring its foundational role in early phonological development (Pan et al., 2016). Conversely, Wang et al. (2023) demonstrated that morphological awareness in 6-year-olds significantly predicted phonological awareness a year later, suggesting a bidirectional relationship between these linguistic skills in Chinese literacy acquisition.

The known cross-linguistic differences in learning to read raises the question of whether bilingual experiences with typologically contrasting languages (Spanish vs. Chinese) influence the longitudinal within-language relations between phonological, morphological and word-reading skills in their heritage language and English.

### 1.2. Word reading in bilinguals

Cross-linguistic transfer models developed for sequential bilinguals (Cummins, 1991) posit that children can effectively transfer skills gained in their first language of proficiency and literacy instruction to advance learning to read in a new or second language of acquisition. However, in heritage-language bilingualism, children’s literacy experiences in the dominant societal language often supersede those of the heritage language. For instance, in the United States, immigrant children predominantly attend schools with English-only literacy instruction and have limited (e.g., family- or community-based) opportunities for developing literacy skills in their heritage language. In such contexts dominated by monolingual education and the known differences in learning to read across languages, it is unclear whether heritage bilingual children develop language-specific paths to literacy in their heritage language and whether cross-linguistic transfer occurs from heritage languages to English, the primary language of reading instruction (Chung et al., 2019).

Therefore, one of the core questions in bilingual heritage-language literacy research is to uncover (a) language-specific paths to literacy development in the heritage language and (b) enduring cross-linguistic transfer influences on the primary language of reading instruction (Chung et al., 2019). To answer these questions, we examine the interrelation between children’s developing *morphological*, *phonological*, and *word-reading* skills in Spanish–English and Chinese–English bilinguals and English monolinguals, in each of their language(s) and as measured over time.

### 1.3. Bilingual transfer in English-dominant settings

Dual-language interactions and cross-linguistic transfer are defining features of bilingual development. Bilingual literacy development frameworks, such as the interactive transfer framework (Chung et al., 2019), specify three key factors influencing dual-language interactions: proficiency, similarity and complexity.

*Proficiency* is critical to consider in bilinguals’ dual-language development. In heritage bilinguals, children often exhibit higher proficiency in their heritage language in early childhood. As bilinguals advance through formal schooling in the dominant language of society, their proficiency profile can flip and thus poses a unique challenge to understanding the bilingual transfer effects (e.g., Durgunoğlu et al., 1993; Wang et al., 2006, 2009). As our study focuses on cross-linguistic differences in bilingual transfer effects, the children needed adequate heritage-language proficiency matched across groups.

*Similarity and complexity* principles further postulate that cross-linguistic transfer is more likely to occur when two bilingual languages share specific relevant features and when such features are either complex or *salient* in one of these languages. In the case of Spanish–English bilinguals, there is a shared alphabetic principle or sound-to-letter mapping, and this feature is more *salient or otherwise has higher predictability in Spanish than in English*. Conversely, in the case of Chinese–English bilinguals, the shared feature is meaning-to-print mapping. Indeed, earlier works have shown that Chinese–English bilinguals may have stronger meaning-to-print associations than either Spanish–English bilinguals or English monolinguals (Hsu et al., 2019; Sun et al., 2022). As Chinese and English are dissimilar in their orthographic and linguistic structure, it is possible that the transfer is achieved *indirectly*, not via direct transfer of morphological awareness but through the overall propensity for stronger reliance on meaning-related cues for visual word recognition (Sun et al., 2022). In this study, we expand this inquiry through a longitudinal approach to examining direct and indirect transfer influences.

### 1.4. Transfer of phonological skills

Phonological awareness skills are generally considered similar across languages and are often found to transfer easily in bilingual learners. Bilingual theories thus commonly view phonological awareness in bilinguals as a single-language-shared set of skills with minimal cross-linguistic differentiation (cf. Chung et al., 2019). Nevertheless, saliency and similarity context also play a role, such that although cross-linguistic transfer of phonological awareness skills is often reported in both Chinese–English (Luo et al., 2014; Sun et al., 2022) and Spanish–English bilinguals (Kelley et al., 2014; Marks et al., 2023; Soto et al., 2019; Sun et al., 2022), the extent of such transfer is stronger in Spanish– English bilinguals (Sun et al., 2022). The difference between the transfers of phonological skills in the two bilingual groups has been attributed to the higher saliency of phonological awareness in Spanish and the stronger similarity between Spanish and English. To date, no study has assessed the longitudinal trajectory of cross-linguistic transfer in heritage bilingual children. Based on the results from these concurrent studies, we predicted substantial longitudinal cross-linguistic transfer of phonological awareness skills in both Spanish–English and Chinese–English bilinguals. However, such transfer would be stronger in the Spanish–English bilinguals.

### 1.5. Transfer of morphological skills

Morphological awareness skills are generally considered more language-specific than phonological skills, as one would need to know the specific lexemes (roots/suffixes) and the grammar rules that govern them to form words (Chung et al., 2019). Therefore, in the bilingual transfer of lexical morphological skills, one must consider both *direct* and *indirect* types of cross-linguistic influence. The direct influence is often measured through associations between literacy skills in languages A and B. For instance, cross-linguistic transfer of morphological awareness is robustly observed between Spanish and English (Kuo et al., 2015; Ramírez et al., 2013), whereas such transfer between Chinese and English is predominantly limited to compound awareness (Pasquarella et al., 2011; Zhang et al., 2010). Cross-linguistic similarity effects play a role here as Spanish and English are two Indo-European languages with shared grammatical affixation rules and common lexical items (e.g., de + scribe), whereas Chinese is structurally and lexically distinct. At the same time, salience may still exert its influence, yielding an *indirect* transfer effect. For instance, Chinese–English bilinguals have been found to form stronger meaning-to-print associations in English relative to English monolinguals or Spanish–English bilinguals, as measured in associations between semantic (vocabulary and lexical morphology) and word-reading skills (Hsu et al., 2019; Ip et al., 2017; Sun et al., 2022).

## 2. The present study

**Figure 1.**
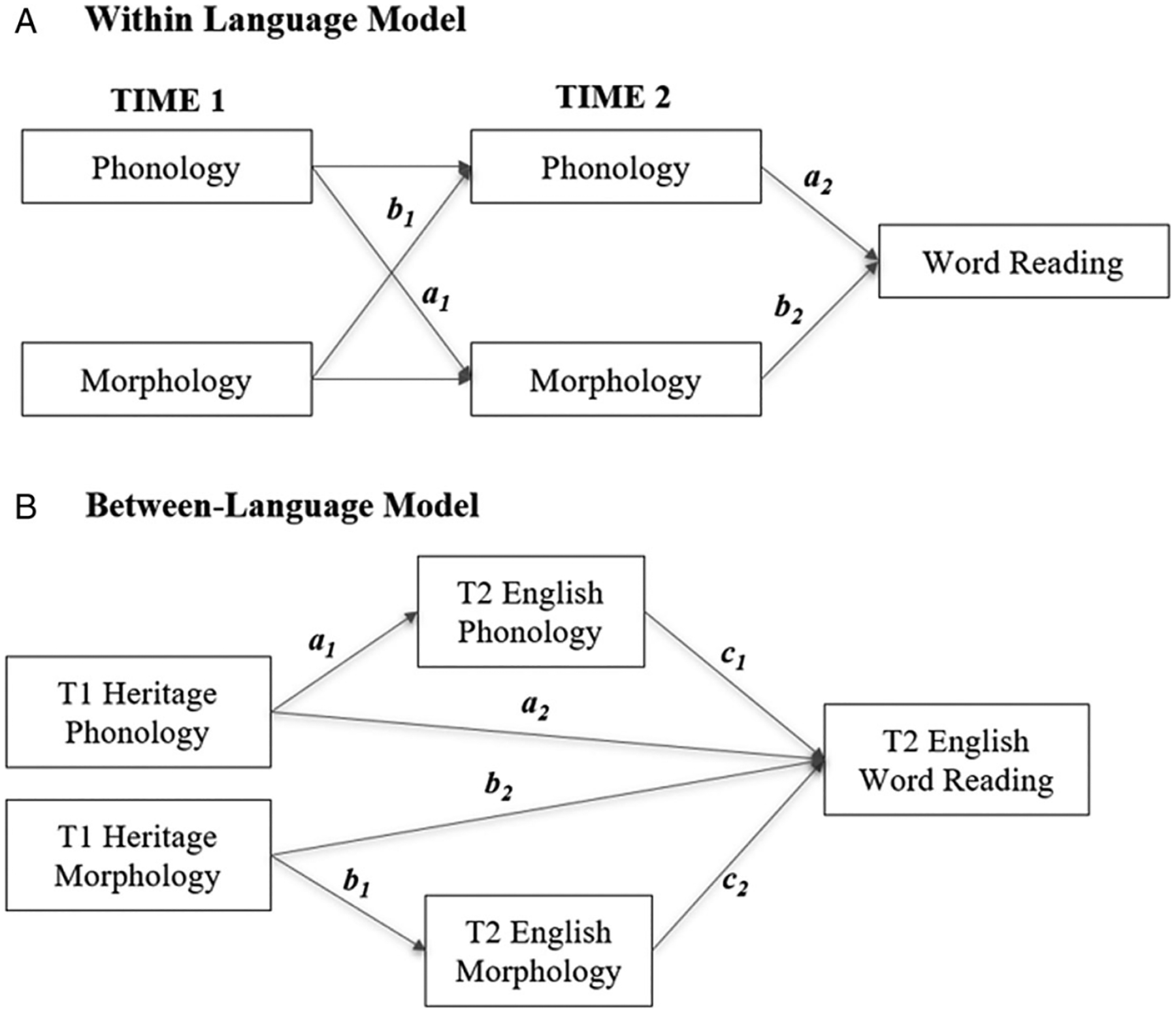
Hypothesized baseline models for research questions 1–3. Panel (A) is the hypothesized within-language CLPM to examine the longitudinal relationship of phonological and morphological awareness and their concurrent relationship to word reading in each of the bilinguals’ languages. For outcomes, see Figures 2 and 3. Panel (B) is the hypothesized between-language SEM model to examine the longitudinal transfer effect from bilinguals’ heritage language to English. For outcomes, see Figure 4.

The present study offers a longitudinal examination of children’s emerging sound-to-print and meaning-to-print associations, as measured through phonological, morphological and word-reading skills in Spanish–English and Chinese–English heritage bilinguals, as well as English monolinguals in each of the children’s language(s). Our first set of questions focused on **cross-linguistic differences between Spanish and Chinese**, the heritage languages of bilingual speakers. We asked: *Do heritage speakers exhibit the same general trends in their word reading as those educated in Spanish- or Chinese-speaking countries?* We examined longitudinal relations between phonological and morphological awareness at time 1 (T1) and word reading at time 2 (T2, one and a half years later). Guided by the extension of the lexical quality framework to cross-linguistic variation and relevant cross-linguistic works, we predicted the following trends. For Spanish, we hypothesized that T1 phonological skills would significantly associate with T2 phonological and morphological skills (Figure 1, panel A, path a1). For Chinese, we hypothesized that T1 morphological awareness would be significantly associated with T2 morphological and phonological skills (Figure 1, panel A, path b1). For the concurrent T2 associations, we further predicted that sound-to-print, or phonological awareness of word reading, associations will be more assertive in Spanish (Figure 1, panel A, path a2), whereas meaning-to-print, or morphological awareness of word reading, associations will be more assertive in Chinese (Figure 1, panel A, path b2).

Our second set of questions focused on bilingual differences in the associations between bilingual speakers’ heritage language and English. The **first bilingual question** focuses on *indirect cross-linguistic transfer effects* at T2, in English. First, we asked: *Are there effects of bilingualism on how children form sound-to-print and meaning-to-print associations in English, their dominant language of literacy instruction?* Guided by the interactive transfer framework and earlier bilingual works, we predicted that bilingual groups would differ from each other at T2 as a function of their heritage-language structure. We predicted that at T2 the strength of sound-to-print concurrent association would be more robust in Spanish–English bilinguals than Chinese–English bilinguals (Figure 1, panel A, a2 in Spanish–English bilinguals > a2 in Chinese–English bilinguals). In contrast, we predicted that the strength of meaning-to-print associations would be more robust in Chinese–English bilinguals than in Spanish–English bilinguals (Figure 1, panel A, b2 in Chinese–English bilinguals > b2 in Spanish–English bilinguals). We also predicted that relative to English monolinguals, Spanish bilinguals would have stronger sound-to-print associations, whereas Chinese bilinguals would have stronger meaning-to-print associations, in English.

Our **second bilingual question** explored the *direct relation* between children’s heritage-language skills at T1 and their English literacy at T2 (Figure 1, panel B). We predicted a direct longitudinal transfer of both phonological and morphological awareness skills in Spanish-language bilinguals, owing to both structural similarity and saliency effects of phonological awareness in Spanish in relation to English. We further predicted that direct longitudinal transfer is more likely to be limited to phonological awareness in Chinese bilinguals.

In sum, the study aimed to shed light on bilingual literacy development by examining direct and indirect transfer influence across Spanish–English and Chinese–English bilinguals. Direct transfer effects were predicted for phonological awareness across both bilingual groups because it is considered a language-general skill. Direct transfer was further predicted for morphological awareness for Spanish–English bilinguals because of the close cross-linguistic distances between the two (shared base and affixes, as well as rules of affixation). In contrast, we thought it would be less likely for Chinese–English bilinguals to exhibit direct morphological transfer effects, due to the substantial differences in lexical structure across the two languages. Nevertheless, we predicted that owing to the salience of morphological characteristics in Chinese literacy, Chinese–English bilinguals may exhibit an indirect transfer of morphology and thus exhibit stronger morphology-to-word-reading associations in English as compared to Spanish–English bilinguals.

## 3. Method

### 3.1. Participants

The sample analyzed comprised of 181 children (48.3% female; *M*_age_ = 7.57; SD = 1.28), predominantly first graders at T1. The participants were recruited from schools and community centers in southeast Michigan, United States. The participants met specific screening criteria, including a minimum standard score of 85 in heritage-language vocabulary and completion of two waves of testing. The final sample included 61 monolingual English children, 60 Spanish–English bilingual children and 60 Chinese–English bilingual children recruited for a larger bilingual literacy study. Descriptive statistics are summarized in Table 1. Consent was obtained in writing from parents, and each child provided assent prior to participating in the study. The Institutional Review Boards of the University of Michigan in Ann Arbor approved the study.

### 3.2. Procedure

Data were collected at two different points in time. The average gap between testing sessions T1 and T2 is 631 days. T1 data were collected through in-person lab testing between May and October 2019. T2 data were collected between December 2020 and July 2021. Due to the COVID-19 pandemic constraints, T2 data were collected virtually on Zoom. Pratt et al. (2022) verified the validity of virtual assessments for language behavior and cognition.

### 3.3. Language and background screenings

At T1, parents completed a Bilingual Language Background and Use Questionnaire. The questionnaire aims to collect information about the children’s cognitive and language development, home language use and exposure, school language use and exposure, physical and mental health history and the parent’s language background. Each participant in the study was either identified as an English monolingual speaker, a Spanish–English bilingual speaker or a Chinese–English bilingual speaker based on their parent’s response to a question about their child’s language background (“Is your child 100% monolingual? Yes/No, If no, what is your child’s second language?”).

At the time of testing, all participants lived in the United States, attended English-only schooling and had age-appropriate English vocabulary and word-reading proficiency. Monolingual English children were exposed to English from birth. Bilingual children were exposed to Spanish or Chinese from birth by at least one parent who was a native speaker of the language and were exposed systematically to English by the age of 2. Parents of bilingual children completed a survey on the hourly input and output of their children’s language usage throughout a typical week. The results showed that Spanish–English bilingual children used Spanish 40% of the week whereas Chinese–English bilingual children used Chinese 38% of the week, with the remaining portion representing time spent using English.

### 3.4. Language and literacy measures in English

At both waves of testing, participants completed a battery of English language and literacy tasks, including standardized measures of phonological awareness, vocabulary, word reading and working memory. The validity and reliability of these tests are described in detail in Sun et al. (2022). The morphological awareness was assessed through an experimental task. Marks et al. (2023) describe the validity and reliability.

#### Phonological awareness.

This was tested with the Elision subtest of the Comprehensive Test of Phonological Processing (CTOPP; Wagner et al., 1997). Children heard a word and were asked to remove a phonetic unit. For example, in English, children were asked to say “farm” without saying /f/ [arm].

#### Morphological awareness.

This was assessed by the Early Lexical Morphology Measure (ELMM) (Marks et al., 2023). Children were given a word and were asked to remove a morphological unit of the word to fit a sentence. For example, in English, “*foggy*. On some mornings, I can see __ [*fog*].” In Spanish, an example is “*Espantapajaros*. A mi me gusta ver los __ [*pajaros*].” ELMM consists of 40 items (15 compounds, 25 derived) and showed a high internal consistency, Cronbach’s *α* > .93 (Marks et al., 2023).

#### Word reading.

This was assessed by the 78-item measure from the letter-word identification subtest of the Woodcock–Johnson IV (Schrank et al., 2014). Children were shown a list of letters and words and were asked to identify single letters and read single words with increased difficulty. Children begin the test from their age-appropriate item and stop when they make six errors. For our current study, the test–retest reliability was .84 for the raw score and .82 for the standard score.

#### Receptive vocabulary.

This was measured with the Peabody Picture Vocabulary Test-5 (PPVT-5, Dunn, 2018). Children heard a word in English and were shown a page with four images. Based on the word the child heard, they pointed to the image that best corresponds to it. Each child starts with their age-appropriate item and stops when they make six consecutive mistakes. The PPVT-5 assessment has a total of 240 items and shows an overall reliability of .97 and a test reliability of .88 (Dunn, 2018). In our current study, the raw score for test–retest reliability was .83 and .71 for the standard score. Although our test–retest reliability is within an acceptable range, the low score of .71 is likely due to numerous contextual factors such as changes from in-person to remote learning and 1.5-year intervals between tests.

#### Working memory: In English, children’s working memory was assessed using a backward digit span task from the Wechsler Intelligence Scale for Children’s edition (Wechsler, 2014).

During the task, children were asked to listen to a series of digits and repeat them in backward order. The first item of the series consisted of two digits, with each subsequent series containing an increasing number of digits.

### 3.5. Language and literacy measures in heritage languages

All bilingual children also completed several heritage-language assessments, including phonological awareness, morphological awareness, vocabulary and word reading across the two time points.

#### Spanish phonological awareness.

In Spanish, phonological awareness was assessed using a 20-item elision measure, the Test of Phonological Processing in Spanish (Francis et al., 2001). For example, given the word “Rojo,” children were then asked to say “Rojo” without saying /r/ [ojo]. The instruction, as well as the ceiling rule, was the same as in the English CTOPP. The internal consistency was .83.

#### Spanish morphological awareness.

We assessed Spanish morphological awareness using a lab-developed measure modeled on the English ELMM task. The Spanish morphology task consisted of 50 items, including 41 derivational words and 9 compound words. The internal reliability for this study was .95.

#### Spanish word reading.

The assessment of Spanish single-word reading was conducted using a standardized measure by a subtest of the Letter Word Identification measure in the Spanish version of Woodcock–Johnson IV–Batería IV Woodcock–Muñoz (Schrank et al., 2005). The test comprises 76 items that require children to identify visually presented letters and read words. The test follows the exact instructions and stopping rule as the English letter-word identification test and has shown high test–retest reliability (>.81, Schrank et al., 2005).

#### Spanish receptive vocabulary.

Spanish receptive vocabulary was measured by a standardized measure, the Spanish version of the PPVT – Test de Vocabulario en Imagenes Peabody (Dunn et al., 1986). The 125-item test has the same instruction and stopping rules as the English PPVT with high reliability (>.90, Dunn, 1986).

#### Chinese phonological awareness.

Chinese phonological awareness was assessed using a self-developed measure based on the same task paradigm as in English and Spanish and a measure from Newman et al. (2011). The assessment consisted of 36 items, including six syllable-level and 30 phoneme-level elision items. For example, a syllable-level elision task asked participants to say “苹果 /ping2 guo3/” without pronouncing the syllable “苹/ping2/” (果, /guo3/), whereas a phoneme-level elision task asked participants to say “和/he2/” without saying the phoneme “/h/” (/e2/). The measure exhibited high internal reliability with the current sample (Cronbach’s alpha = .85).

#### Chinese morphological awareness.

Chinese morphological awareness was assessed through a morphological construction task, modified based on a measure from Song et al. (2015). Children were asked to create a new compound word based on the morphemes in the given word. For example, “Apple trees grow apples. What trees might grow bread?” (bread-trees). “一颗长苹果的树, 我们叫它苹果树, 一棵会长出面包的树我 们叫它?” (面包树). The task comprised of 25 items and demonstrated high internal reliability (Cronbach’s alpha = .94).

#### Chinese character recognition and reading.

The experimental measure for Chinese word recognition and the reading task was developed based on the Chinese curriculum (MLP Chinese and Jida Chinese Academy) commonly used in heritage Chinese schools. The measure comprised of two components: character recognition and character reading. The character recognition component included 30 items that required participants to select the correct character that was read to them. The character reading component consisted of 60 items that asked children to read aloud the displayed characters. The measure exhibited high internal reliability (Cronbach’s alpha = .81).

#### Chinese receptive vocabulary.

This was measured by the Chinese version of the PPVT – PPVT-R (Lu & Liu, 1998). The measure has 125 items with the same instruction as in English PPVT. The test–retest reliability was reported as .84 (Lu & Liu, 1998).

### 3.6. Analytical plan

To address the three research questions, we employed cross-lagged panel models (CLPMs) to analyze the longitudinal temporal dynamic relationship among phonological awareness, morphological awareness and word-reading skills in bilinguals and monolinguals, and structural equation modeling (SEM) to examine the longitudinal relationship between phonological awareness and morphological awareness in heritage language and English. For this purpose, the analysis used the lavaan package in R (Rosseel, 2012). The full information maximum-likelihood method was used to handle missing data, and the maximum-likelihood robust estimator was employed to estimate the model parameters. Model fit was evaluated using multiple indices, including model Chi-square (*χ*^2^), comparative fit index (CFI) > .90, standardized root mean square residual (SRMR) < .08 and root mean square error of approximation (RMSEA) < .05 (Kline, 2015).

## 4. Results

Children’s performance on all the English- and heritage-language tasks at both T1 and T2 are presented in Table 2. All three groups showed comparable English proficiencies, except for T1 vocabulary. Pairwise *t*-tests showed that the monolingual group had a higher average English vocabulary than the bilingual groups, whereas the two bilingual groups showed a comparable English vocabulary level. From T1 to T2, all children demonstrated performance growth in all measures. Pairwise *t*-tests showed that T2 performances were significantly better than T1 in raw scores for all tasks across languages, all *p*’s < .001. To examine the relations among tasks, times and languages, we conducted a partial correlation analysis controlling for the effects of age, working memory, maternal education and vocabulary (Table 3). All English tasks were significantly correlated with one another across both time points and groups (*r* = .25–.77). Heritage-language tasks for bilinguals also showed significant correlations with one another across times (*r* = .27–.79). Spanish bilingual children’s most Spanish task performances also significantly correlated with their English task performances. Spanish morphological awareness was only significantly correlated with T1 English morphological awareness, word reading and T2 English morphological awareness.

In contrast, fewer Chinese tasks showed significant correlations with English tasks. Chinese phonological awareness was significantly correlated with all English tasks. However, Chinese morphological awareness only significantly correlated with T1 English morphological awareness, word reading, T2 English vocabulary and word reading.

### 4.1. Research question 1: longitudinal relationships of phonological awareness, morphological awareness and word reading in heritage languages

To address research question 1, we examined the longitudinal relationships of phonological, morphological awareness and word reading. We conducted two CLPMs (one Spanish heritage model and one Chinese heritage model) to examine the longitudinal relationship of phonological and morphological awareness skills at T1 as well as word-reading skills in the heritage language at T2. Both models controlled for age, maternal education, working memory and receptive vocabulary.

**Figure 2.**
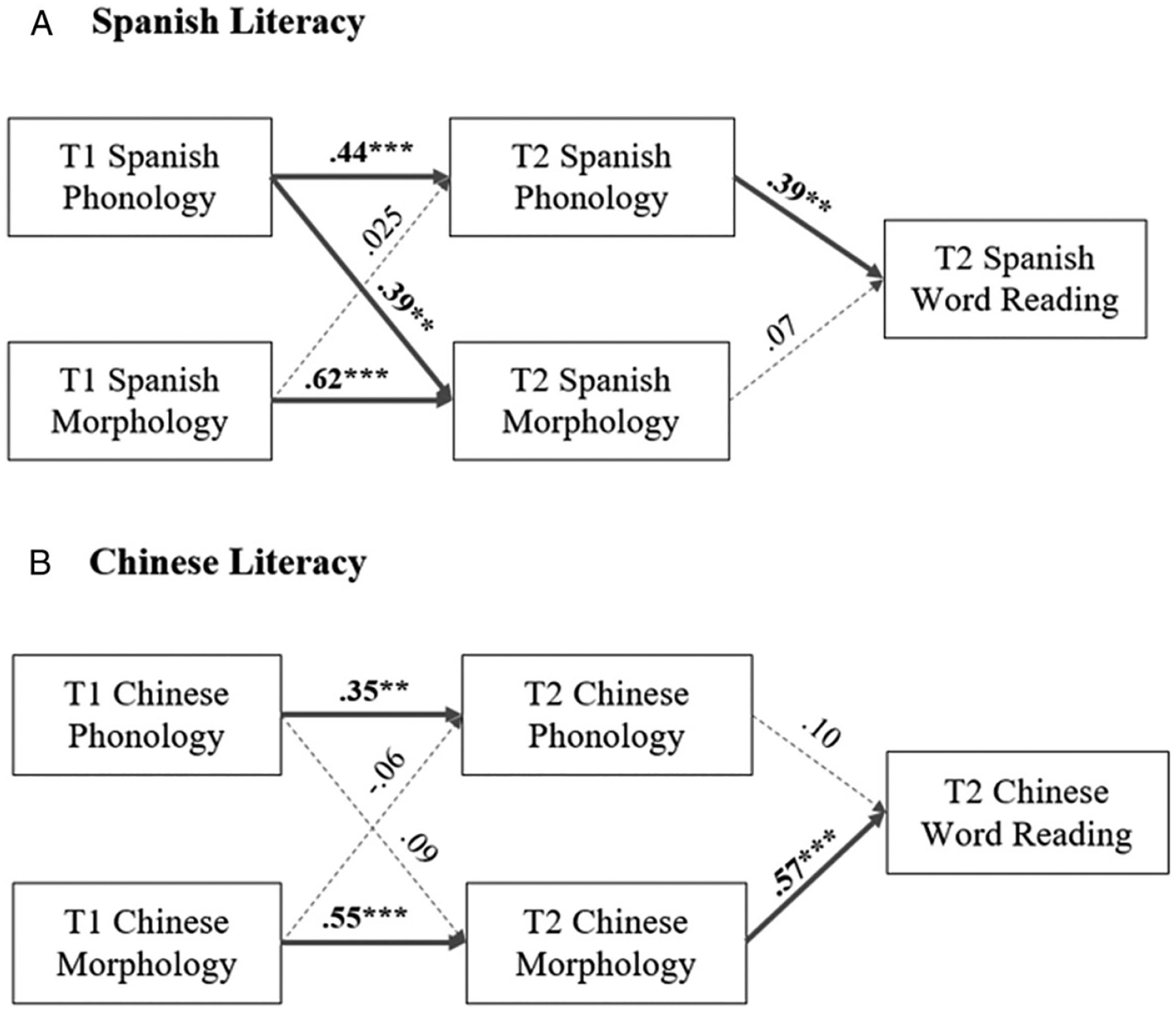
CLPMs for word reading in (A) Spanish- and (B) Chinese-heritage languages of the bilingual groups. All models controlled for age, working memory, maternal education and receptive vocabulary. All coefficients were standardized. *p < .05; **p < .01; ***p < .001.

The Spanish heritage model suggests very good fit (*χ*^2^ = 5.16, *p* = .43, CFI = .97, RMSEA = .019, SRMR = .012) (Figure 2). T1 phonological awareness predicted T2 morphological awareness (*β* = .39, *p* < .01). The autoregressive paths between the two time points for phonological (*β* = .44, *p* < .001) and morphological awareness (*β* = .62, *p* < .001) were significant. T2 phonological awareness significantly predicted word reading (*β* = .39, *p* < .05).

The Chinese heritage model also suggests a very good fit (Chinese: *χ*^2^ = 4.98, *p* = .51, CFI = 1.00, RMSEA = .018, SRMR = .011) (Figure 2). No significant cross-lagged relations between T1 and T2 phonological and morphological awareness were observed. The autoregressive paths between two time points for phonological (*β* = .35, *p* < .01) and morphological awareness (*β* = .55, *p* < .001) were significant. T2 morphological awareness significantly predicted T2 word reading (*β* = .57, *p* < .001).

### 4.2. Research question 2: longitudinal relationships of phonological awareness, morphological awareness and word reading in English (indirect transfer)

**Figure 3.**
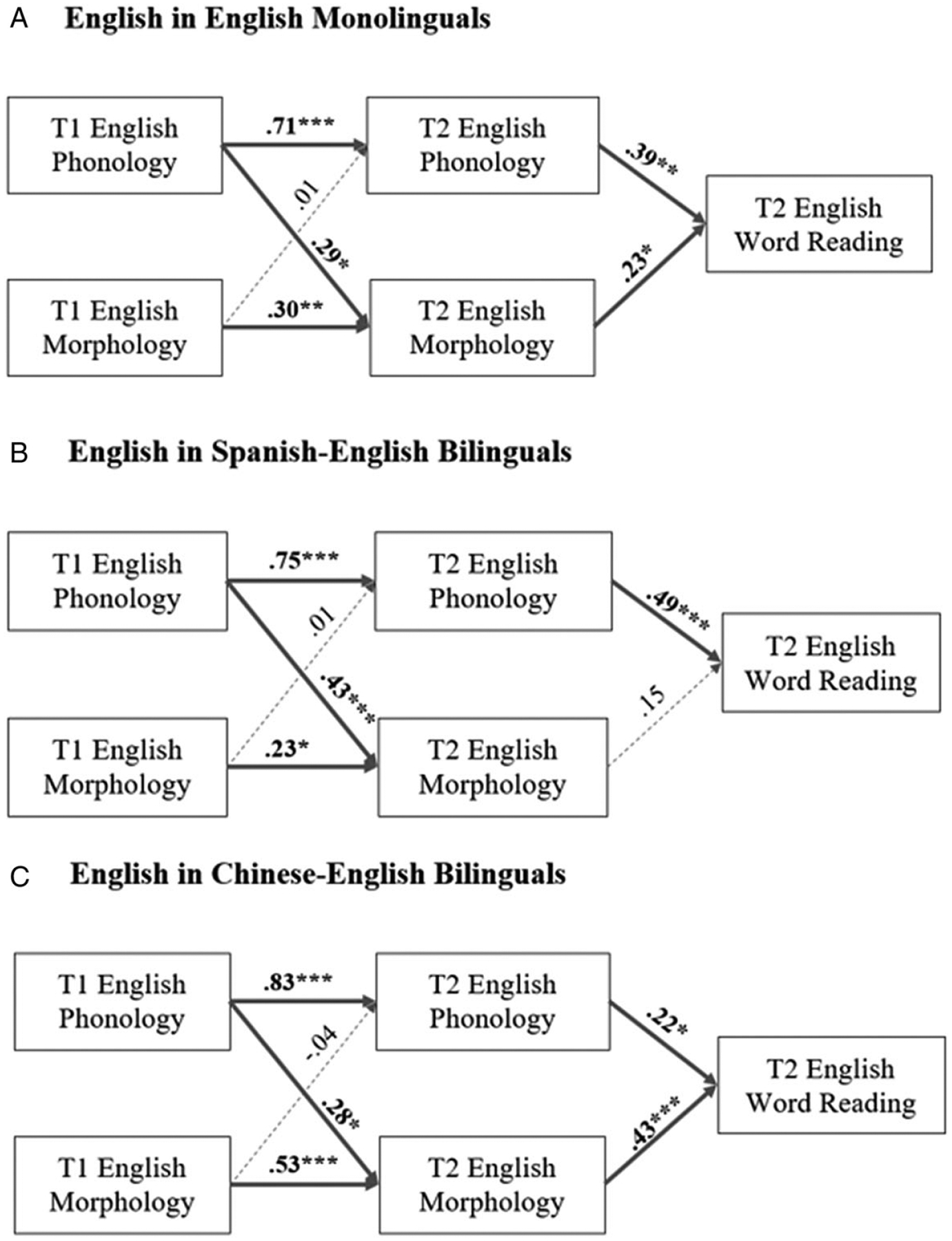
CLPMs for word reading in English in (A) Spanish–English bilinguals, (B) Chinese–English bilinguals and (C) English monolinguals. All models are controlled for age, working memory, maternal education and receptive vocabulary. All coefficients were standardized. *p < .05; **p < .01; ***p < .001.

The second aim of the study was to understand the cross-linguistic transfer effect. To examine the indirect transfer effect, we conducted CLPM analyses on Spanish–English bilingual, Chinese–English bilingual and English monolingual children’s shared language – English. All models controlled for age, maternal education, working memory and receptive vocabulary. Before analyzing the structural CLPM, we first tested model invariance by fitting configural, strong and strict invariance models based on Kline (2005) and Rosseel (2012). In this step, we first fitted a configural model without imposing cross-group equality constraints and intercept. Next, we fitted a strong invariance model in which intercept equality was imposed across the three groups. Finally, we fitted a strict invariance model in which we imposed constraints on error variances and covariances across groups. The model comparison statistics suggested strong measurement invariance. The final model (Figure 3) showed a very good fit (*χ*^2^ = 5.08, *p* = .37, CFI = .98, RMSEA = .02, SRMR = .014).

For Spanish bilinguals, T1 English phonological awareness significantly predicted T2 morphological awareness (*β* = .33, *p* < .001). Both phonological and morphological awareness showed significant autoregressive effects between the two time points (phonology: *β* = .75, *p* < .001; morphology: *β* = .23, *p* < .05). T2 English phonological awareness significantly predicted T2 English word reading (*β* = .49, *p* < .05).

For Chinese bilinguals, T1 English phonological awareness also significantly predicted T2 morphological awareness (*β* = .28, *p* < .05). Similar autoregressive effects were observed for phonological awareness (*β* = .83, *p* < .001) and morphological awareness (*β* = .53, *p* < .001). Both T2 phonological and morphological awareness significantly contributed to T2 word reading (phonology: *β* = .22, *p* < .05; morphology: *β* = .43, *p* < .001).

For English monolinguals, T1 phonological awareness significantly contributed to T2 morphological awareness (*β* = .29, *p* < .05). Significant autoregressive effects were observed for phonological and morphological awareness across the two time points (phonology: *β* = .71, *p* < .001; morphology: *β* = .30, *p* < .05). Both T2 phonological and morphological awareness significantly contributed to T2 word reading (phonology *β* = .39, *p* < .001; morphology: *β* = .23, *p* < .05).

To statistically test whether Spanish–English bilingual children have a stronger sound-to-print association compared to English monolinguals, we conducted model comparisons (Figure 1, panel A, paths a_1_ and a_2_). First, we compared our final model with a separate model constraining T1 phonological awareness to T2 morphological awareness coefficients (Figure 1, panel A, path a_1_) to be equal across the two groups. The test statistics indicated a significant difference between the constrained and unconstrained model ($\chi_{{\text{diff}}^{2}}\left(1\right)=6.37$, *p* = .03). Therefore, the contribution of T1 English phonological awareness to T2 English morphological awareness in Spanish–English bilinguals was significantly stronger than English monolinguals. Similarly, we also conducted a model comparison constraining the T2 English phonological awareness to the T2 English word-reading path (Figure 1, panel A, path a_2_). The results showed a significant difference between the constrained and unconstrained model ($\chi_{{\text{diff}}^{2}}\left(1\right)=4.98$, *p* = .02), indicating phonological awareness of Spanish–English bilingual plays a stronger role in English word reading compared to English monolinguals.

Finally, to statistically test whether Chinese–English bilinguals have a stronger meaning-to-print association than English monolinguals, we conducted model comparisons (Figure 1, panel A, path b_2_). We compared our final model with a separate model constraining T2 English morphological awareness to T2 English word-reading coefficients to be equal across the two groups. The test statistics indicated a significant difference between the constrained and unconstrained models ($\chi_{{\text{diff}}^{2}}\left(1\right)=8.27$, *p* = .01), indicating the contribution of T2 English morphological awareness to T2 English word reading in Chinese–English bilinguals was significantly stronger than the English monolingual group.

### 4.3. Research question 3: longitudinal transfer of heritage-language skills to English literacy development (direct transfer)

**Figure 4.**
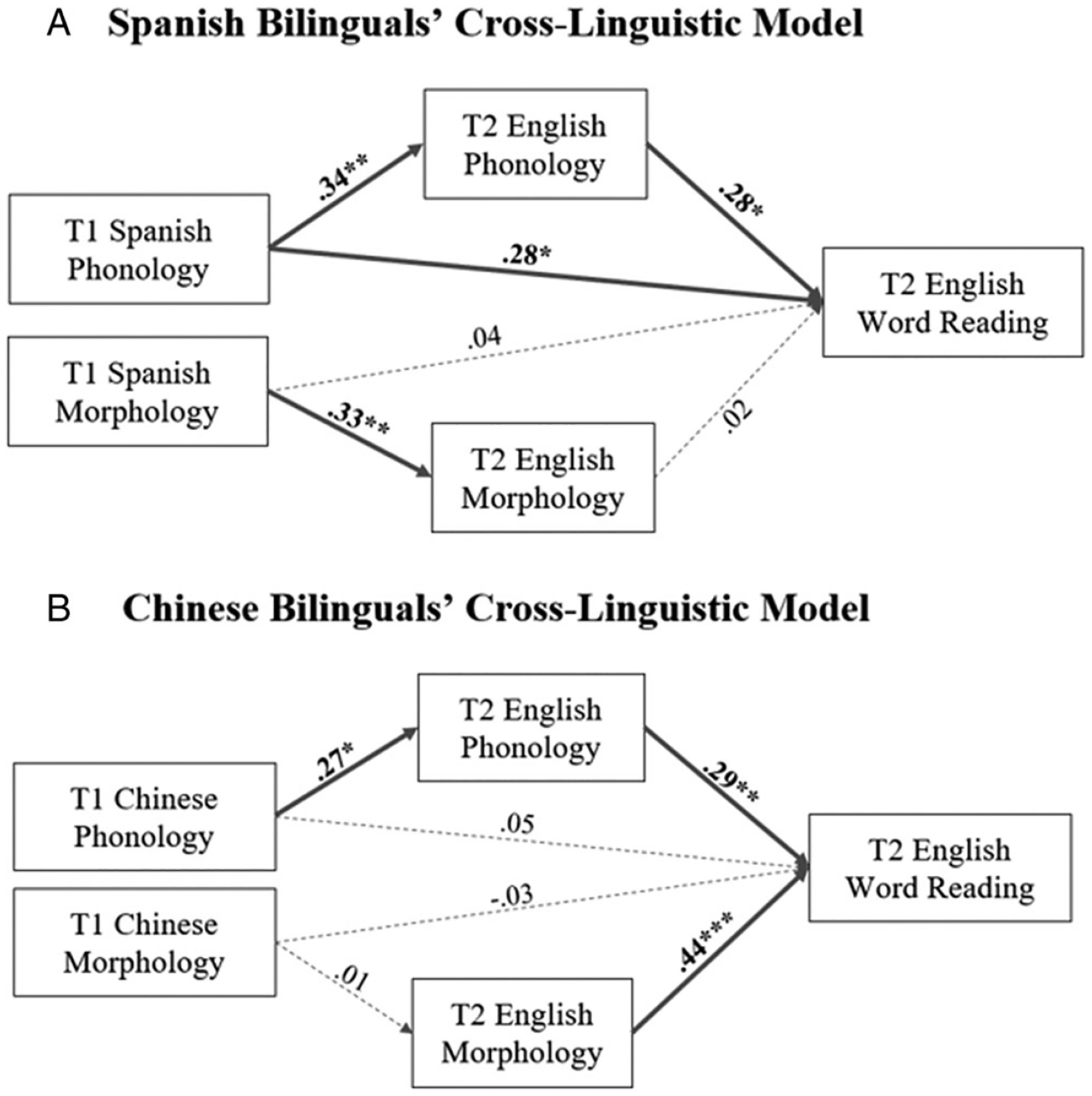
Cross-linguistic SEM models that examine associations between both heritage- and English-language skills in relation to English word reading in bilinguals. Models are controlled for T1 English vocabulary, T1 English phonological awareness, T1 English morphological awareness, age, working memory and material education. All coefficients were standardized. *p < .05; **p < .01; ***p < .001.

Our third research question investigated the direct longitudinal transfer effect from bilinguals’ heritage language to English literacy skills. To answer this question, we conducted SEM analyses, including bilinguals’ T1 heritage phonological and morphological awareness, T2 English phonological and morphological awareness and T2 English word reading. In our SEM models, T2 phonological and morphological awareness and T2 English word reading were modeled as endogenous variables, whereas heritage T1 phonological and morphological awareness were modeled as exogenous variables. We included T1 English phonological and morphological awareness in our analysis to control for the autoregressive effect. Additionally, we controlled for age, working memory, maternal education and T1 English vocabulary. We employed the same model invariance testing described previously, which suggested strict invariance. Our final model (Figure 4) demonstrated a very good fit (Spanish: *χ*^2^ = 9.89, *p* = .31, CFI = .97, RMSEA = .034, SRMR = .013; Chinese: *χ*^2^ = 10.28, *p* = .26, CFI = .98, RMSEA = .018, SRMR = .012).

In Spanish bilinguals, T1 Spanish phonological and morphological awareness significantly contributed to T2 English phonological (*β* = .31, *p* < .05) and morphological awareness (*β* = .31, *p* < .05) respectively, above and beyond the autoregressive effect. T1 Spanish phonological awareness also significantly contributed to T2 English word reading (*β* = .28, *p* < .05).

In Chinese bilinguals, only T1 Chinese phonological awareness significantly contributed to T2 English phonological awareness (*β* = .27, *p* < .05), above and beyond the English phonological awareness autoregressive effect. At T2, similar to the results for research question 2, both English phonological and morphological awareness significantly contributed to English word reading (phonology: *β* = .29, *p* < .01; morphology *β* = .44, *p* < .001).

## 5. Discussion

The study asked how dual-language literacy skills contribute to young bilinguals’ literacy development over time. To answer this question, we examined phonological, morphological and word-reading skills in Spanish–English and Chinese–English heritage-language speakers and English monolinguals across two time points separated by about a year-and-a-half. Guided by the interactive transfer framework (Chung et al., 2019), we hypothesized that bilingual literacy transfer varies as a function of literacy skill and cross-linguistic distances. Close examination of children’s morphological literacy skills offers the strongest support for this hypothesis. Morphological awareness skills are more language specific, and although they are more closely related in Spanish and English, it is in Chinese where they exert the most influence in learning to read. We therefore predicted that although Spanish–English bilinguals may exhibit stronger direct transfer effects between their morphological awareness skills in their two languages, it would be Chinese–English bilinguals who would exhibit stronger indirect transfer effects in their English literacy (i.e., stronger morphology-to-word-reading associations in English relative to other bilingual groups). Our data support this hypothesis. In particular, although both bilingual groups showed direct transfer of phonological awareness skills from heritage language at T1 to English at T2, only Spanish bilinguals demonstrated this effect for morphological awareness. Nevertheless, Chinese bilinguals showed stronger morphological awareness to word-reading associations in English at T2. In contrast, Spanish bilinguals showed this effect for phonological awareness. These results contribute to literacy theory by illuminating the factors that influence cross-linguistic interactions and support the literacy development that support heritage-language bilinguals’ literacy development over time.

### 5.1. Heritage-language literacy development: Spanish and Chinese

Our heritage-language findings are generally consistent with decades of monolingual literacy research in Spanish and Chinese (D’Alessio et al., 2019; Kim & Pallante, 2012; Lázaro et al., 2021). Earlier works with Spanish monolinguals and English monolinguals suggest that phonological skills may support the development of word segmentation skills essential for further development of both phonological and morphological skills over time (e.g., Lázaro et al., 2021). Consistently, in the Spanish heritage-language model (Figure 1A), we found that phonological skills at T1 were directly associated with both phonological and morphological skills at T2 (whereas morphological skills at T1 were only associated with morphological skills at T2). Moreover, at T2, word reading was associated with concurrent phonological awareness.

Recent work in Chinese monolinguals suggests that morphological awareness contributes to both morphological and phonological awareness development in early grades (Wang et al., 2023). In contrast, in the Chinese heritage-language model (Figure 2B), we found that morphological skills at T1 were associated only with morphological skills at T2, whereas phonological skills at T1 were associated with phonological skills at T2. The absence of a cross-lagged relation between T1 morphological skills and T2 phonological skills in Chinese–English bilinguals may stem from greater heterogeneity in learning Chinese as a heritage language (compared to monolingual learning) as well as from cross-linguistic transfer from English, which may contribute to strengthening of phonological literacy skills. However, consistent with monolingual studies suggesting that morphological awareness plays a central role in literacy development in Chinese, we found that at T2, word reading was associated with morphological awareness in Chinese. Notably, participants in this study attended English-only schools, complemented by limited heritage-language instruction (such as weekend heritage-language schools). Thus, our results show that even limited exposure to heritage-language literacy instruction supports the development of language-specific literacy pathways in young bilinguals.

### 5.2. English literacy: in bilinguals and monolinguals

Our next step was to examine similarities and differences in English literacy development among Spanish–English bilinguals, Chinese–English bilinguals and English monolinguals. English was the primary language of academic instruction for all children. Across time points, the three groups had similar phonological awareness and word-reading proficiency. However, monolinguals had better English vocabulary and morphological awareness than the bilinguals, who were similar. Nevertheless, as the Spanish and Chinese bilinguals showed principled group differences in their sound-to-print associations both in comparison with the monolinguals and between each other, we suggest that the observed trends were more likely to stem from cross-linguistic influences than group differences in vocabulary or morphological awareness between the groups. Earlier works have also found that bilingual children’s academic vocabularies are comparable to those of their monolingual counterparts. Yet, their knowledge of non-academic words may be more robust in their heritage language. This distinction may therefore have influenced their performance on our interrelated vocabulary and morphological semantic tasks (Bialystok et al., 2010).

Longitudinally, all three groups demonstrated skill associations and T1 phonological and T2 morphological awareness associations (Figure 3, panels A–C). A direct comparison between the groups revealed that the association between phonological awareness at T1 and morphological awareness at T2 was stronger in Spanish–English bilinguals relative to the other readers. Cross-sectionally, at T2, phonological and morphological awareness were both associated with word reading in monolinguals and Chinese bilinguals. In contrast, only phonological awareness was associated with word reading in Spanish–English bilinguals. Moreover, the phonological awareness-to-word-reading association was stronger in Spanish–English bilinguals compared to the other groups. In contrast, the morphological awareness-to-word-reading association was stronger in Chinese bilinguals.

These longitudinal observations confirm and extend results from earlier cross-sectional studies in similar populations (Hsu et al., 2019; Kremin et al., 2019; Sun et al., 2022). Consistent with our results, Chinese–English bilingual children have been reported to have a stronger concurrent association between morphological skills and word-reading skills in English than English monolinguals (Hsu et al., 2019), whereas Spanish–English bilingual children have been reported to have a stronger concurrent relationship in word reading than English monolinguals (Kremin et al., 2019). Sun et al. (2022) replicated these findings in directly comparing Spanish bilinguals, Chinese bilinguals and English monolinguals. The present study extends previous concurrent findings by showing that cross-linguistic observations have enduring longitudinal effects.

### 5.3. English literacy: a dual-language model

The present study examined longitudinal relations between bilinguals’ heritage-language literacy skills during the initial test (T1) and their English literacy skills about a year-and-a-half later (T2).

Bilingualism theories suggest that phonological awareness is a shared literacy skill across languages, making it the skill that can easily transfer from one language to another (Chung et al., 2019). Indeed, we found that T1 heritage-language phonological awareness skills made significant contributions to the phonological awareness skills in English at T2, above and beyond T1 English phonological skills. This finding aligns with previous longitudinal research on cross-linguistic transfer of phonological awareness in Spanish–English bilinguals (Kelley et al., 2014; Lindsey et al., 2003; Páez & Rinaldi, 2006) and Chinese–English bilinguals (Luo et al., 2014; Wang et al., 2005). For instance, Luo et al. (2014) found longitudinal transfer, a direct bidirectional transfer of phonological awareness skills between bilinguals in Chinese and English. The direct transfer effect of phonological awareness can be attributed to the universality of this skill across languages. This effect may be even more pronounced in Spanish–English bilinguals due to the salient role of phonological awareness in learning to read in Spanish, further reinforcing this transfer.

Morphological structure is generally considered the type of language and literacy skill that is more language-specific and, therefore, harder to transfer across languages, especially linguistically distant languages (Chung et al., 2019). Indeed, only the Spanish–English bilinguals showed a significant association between T1 heritage-language morphological awareness skills and T2 morphological awareness skills in English. The Spanish group findings are generally consistent with earlier works on concurrent cross-linguistic associations as both Spanish and English are Indo-European languages with many shared Latinate words and overall linguistic structure (Ramirez et al., 2010, 2013; Sun et al., 2022). A more complex picture of cross-linguistic transfer of morphology is observed in Chinese bilinguals. Although both compound and derivational morphologies exist in English and Chinese, their distribution and structural characteristics differ markedly between the two languages. Consequently, we find weaker associations between Chinese and English morphology in Chinese than in Spanish bilinguals. Nevertheless, we also find a stronger association between morphological awareness and reading in English in the Chinese than Spanish bilinguals, an observation that we attribute to the cross-linguistic transfer of overall reading strategies in Chinese where morphology plays a key role in visual word recognition.

In light of the longitudinal nature of our inquiry, it is important to note that morphological skills become more important in English and other alphabetic languages when children grow older. The participants here were in the early stage of reading acquisition (Carlisle & Kearn, 2017; Roman et al., 2009; Singson et al., 2000). It is therefore possible that the types of cross-linguistic transfer that Spanish and Chinese bilinguals enjoy may diverge even further as they get older. Continued simultaneous growth in Spanish and English is likely to afford bilingual speakers of these languages a remarkable continued boost in their vocabulary, lexical morphology and recognition of cognates in speech and in print as Latinate words come to dominate academic English in later grades (Cunningham & Graham, 2000). In Chinese, advanced literacy is also associated with advanced knowledge of derivational morphology, a complex low-frequency feature of Chinese. Therefore, it is more likely that children’s advancements in higher-order metacognitive skills of being able to work with complex linguistic units would be the key driving factor in cross-linguistic transfer at more advanced levels of bilingual Chinese–English literacy (Chung et al., 2019).

Taken together, the longitudinal cross-linguistic transfer effects appear to be substantially influenced by both the cross-linguistic similarity of the literacy skill constructs (in the case of phonological awareness) as well as the similarity/distances between the bilinguals’ two languages (in the case of morphological awareness).

## 6. Theoretical implications

Cross-linguistic perspectives on literacy development have emphasized the complexity of bilingual literacy phenomena as they come under the influence of multiple factors relating to children’s dual-language proficiency with core characteristics of their orthographies and the cross-linguistic distances between them. Guided by the interactive transfer framework (Chung et al., 2019) and leveraging the cross-linguistic distances between Spanish and Chinese, we honed in on Spanish–English and Chinese–English bilinguals’ emerging phonological and morphological awareness skills – and how they change over time. Our findings offer a powerful testament to the complexity of cross-linguistic transfer phenomena and the plasticity of bilingual children’s emerging lexical systems. On the one hand, cross-linguistic similarities can help reciprocally support the development of similar and potentially shared metalinguistic literacy skills, such as phonological awareness across most languages and morphological awareness for structurally similar languages (e.g., Spanish and English). On the other, certain high saliency factors, notably lexical morphology within Chinese, appear to be able to transcend what seems like miles of cross-linguistic distance in both spoken and written word structure in Chinese and English. This effect manifests as stronger associations between morphological skill and single-word reading in English among Chinese–English bilinguals, when compared with English monolinguals and Spanish–English bilinguals. These findings suggest a degree of variability and plasticity in how emerging readers build their lexical representations and how bilingual experiences with structurally distinct languages can effectively influence this plasticity – with two languages offering mutual support of the emerging literacy skills.

## 7. Limitations

We acknowledge several limitations in the present study. First, although the measures across English, Spanish, and Chinese were carefully matched, it is important to note that the morphological awareness task assessed different types of morphological awareness across languages. Although the English and Spanish tasks focused on derived word segmentation and manipulation, the Chinese task targeted compound awareness due to the language’s prevalent compounding structure. Thus, task-related differences may also influence our findings. Second, the study’s power was limited in providing a comprehensive examination of longitudinal development and transfer effects across different age groups. Third, the study sample predominantly comprised of individuals from mid-to-high socioeconomic status households. Therefore, future research with more diverse and representative samples of a similar age range is necessary to establish the robustness and applicability of these findings. Last but not least, we must also acknowledge that the correlational nature of the analysis limits the inferences from this study. Although our findings suggest associations with language skills, these are open to alternative interpretations.

Furthermore, we acknowledge that using traditional CLPMs has its limitations. Specifically, the CLPM does not distinguish between within-person effects and between-person effects. Our study, with longitudinal data collected at only two time points, was constrained to using this model. Future research with more waves of data and possibly employing other cross-lagged panel analyses (such as random intercept CLPMs) would be invaluable in providing a more nuanced understanding of these relationships and verifying the effects observed in the current study.

## 8. Conclusions

The present study explored the role of dual-language skills in children’s literacy development over time. The study investigated phonological, morphological and word-reading abilities in Spanish–English, Chinese–English heritage-language bilinguals and English monolingual children at two separate time points with approximately a year-and-a-half gap. The results revealed language-specific trends in children’s heritage-language development: Spanish bilinguals demonstrated more robust associations between phonological awareness and word reading. In comparison, Chinese bilinguals exhibited stronger associations between morphological awareness and word reading in their respective heritage languages. Importantly, these language-specific trends also influenced children’s reading in English, which is their shared primary academic language. Although bilingual and monolingual groups showed somewhat different patterns of English literacy development, all groups demonstrated robust longitudinal associations between their sound- and meaning-based literacy skills, suggesting that literacy instruction that includes systematic phonological, morphological and orthographic training is critical for bilingual and monolingual speakers (Goldenberg, 2023). The findings further suggest that supporting heritage-language literacy may further strengthen emerging bilinguals’ literacy development across their languages.

## Figures and Tables

**Table 1.** Participants’ demographics

	Full sample	Chinese bilinguals	Spanish bilinguals	English monolinguals	*F*	*p*	Group difference
*N*	181	60	60	61			
Gender %Females	48.3	45	50	45			
Time 1, age *M* (SD)	7.54 (1.25)	7.25 (1.36)	7.68 (1.18)	7.55 (1.17)	1.84	.162	C = S = E
Time 2, age *M* (SD)	9.35 (1.35)	9.12 (1.44)	9.47 (1.29)	9.15 (1.24)	1.28	.281	C = S = E
T1 - T2 gap *M* (SD), days	663 (86)	680 (67)	655 (94)	586 (103)	17.8	<.001	C = S > E

C, Chinese; S, Spanish; E, English.

**Table 2.** Language and literacy performance (means and standard deviations) for Spanish–English bilinguals, Chinese–English bilinguals and English monolinguals

	Spanish bilinguals *M* (SD)	Chinese bilinguals *M* (SD)	English monolinguals *M* (SD)	*F*-value	*p*-value
English tasks (standard scores)					
T1 phonological awareness	10.97 (3.07)	11.47 (2.65)	11.08 (2.46)	.67	.512
T2 phonological awareness	10.80 (2.85)	11.45 (2.79)	10.64 (2.82)	1.70	.193
T1 word reading	110.88 (18.50)	113.58 (14.75)	111.61 (11.53)	.75	.482
T2 word reading	110.00 (16.27)	116.68 (13.49)	113.75 (11.15)	2.81	.071
T1 vocabulary	100.68 (19.80)	105.48 (19.43)	117.59 (15.54)	9.15	<.001
T2 vocabulary	107.48 (18.45)	113.97 (19.67)	118.72 (13.52)	3.93	.023
English tasks (raw scores)					
T1 morphological awareness	24.66 (13.82)	27.05 (16.93)	32.03 (15.39)	2.89	.062
T2 morphological awareness	41.90 (9.76)	44.67 (11.75)	47.03 (9.92)	2.67	.067
T1 phonological awareness	22.25 (7.60)	22.02 (7.55)	22.67 (6.52)	.04	.962
T2 phonological awareness	26.65 (5.97)	27.15 (5.18)	26.10 (5.64)	.87	.424
T1 word reading	45.47 (15.22)	46.00 (17.15)	47.79 (13.41)	.21	.823
T2 word reading	58.73 (9.19)	60.12 (9.88)	59.39 (8.75)	.44	.652
T1 vocabulary	138.92 (28.27)	139.14 (34.36)	159.12 (23.14)	6.74	.002
T2 vocabulary	168.85 (23.51)	170.73 (26.55)	179.63 (18.94)	1.63	.198
Heritage tasks (Spanish/Chinese)					
T1 phonological awareness	13.03 (6.31)	19.70 (10.82)			
T2 phonological awareness	15.58 (5.16)	27.57 (5.95)			
T1 morphological awareness	28.16 (12.50)	13.07 (6.69)			
T2 morphological awareness	30.23 (7.23)	17.26 (5.64)			
T1 word reading	41.07 (20.68)	34.08 (22.24)			
T2 word reading	47.70 (18.01)	47.74 (21.37)			
T1 vocabulary	66.78 (17.58)	56.93 (19.98)			
T2 vocabulary	78.88 (18.80)	64.96 (23.84)			

**Table 3.** Partial correlations among English- and heritage-language (Chinese/Spanish) tasks by language group

English monolinguals	2	3	4	5	6	7	8	9	10	11	12	13	14	15	16
English															
1. T1 phonological awareness	**.53**	**.46**	**.32**	**.33**	**.36**	**.50**	**.57**								
2. T2 phonological awareness	-	**.11**	**.46**	**.33**	**.47**	**.42**	**.57**								
3. T1 morphological awareness		-	**.24**	**.24**	**.34**	**.48**	**.28**								
4. T2 morphological awareness			-	**.32**	**.66**	**.54**	**.55**								
5. T1 vocabulary				-	**.56**	**.28**	**.34**								
6. T2 vocabulary					-	**.32**	**.46**								
7. T1 word reading						-	**.72**								
8. T2 word reading							-								
Chinese bilinguals	2	3	4	5	6	7	8	9	10	11	12	13	14	15	16
English															
1. T1 phonological awareness	**.78**	**.54**	**.52**	**.28**	**.25**	**.63**	**.51**	**.68**	**.37**	**.33**	.23	.06	−.15	.09	.21
2. T2 phonological awareness	-	**.47**	**.48**	**.29**	**.25**	**.58**	**.57**	**.61**	**.51**	.20	**.25**	.11	.01	.18	**.25**
3. T1 morphological awareness		-	**.61**	**.57**	**.46**	**.47**	**.49**	**.56**	**.33**	**.45**	**.34**	.20	.09	.16	.20
4. T2 morphological awareness			-	**.47**	**.42**	**.59**	**.65**	**.34**	.15	.19	.17	.18	−.03	.01	.06
5. T1 vocabulary				-	**.67**	**.50**	**.48**	**.34**	**.25**	.12	.21	.15	.00	.04	.18
6. T2 vocabulary					-	**.41**	**.35**	**.30**	**.30**	.22	**.35**	.14	.13	.20	**.25**
7. T1 word reading						-	**.79**	**.57**	**.41**	**.28**	**.30**	.03	−.19	.02	**.26**
8. T2 word reading							-	**.38**	**.40**	**.29**	**.33**	.06	−.06	.07	**.27**
Chinese															
9. T1 phonological awareness								-	**.55**	**.54**	**.39**	**.33**	**.27**	**.50**	**.58**
10. T2 phonological awareness									-	**.40**	**.50**	**.35**	**.45**	**.36**	**.51**
11. T1 morphological awareness										-	**.67**	**.57**	**.53**	**.51**	**.52**
12. T2 morphological awareness											-	**.54**	**.60**	**.51**	**.67**
13. T1 vocabulary												-	**.74**	**.36**	**.45**
14. T2 vocabulary													-	**.57**	**.61**
15. T1 word reading														-	**.79**
16. T2 word reading															-
Spanish bilinguals	2	3	4	5	6	7	8	9	10	11	12	13	14	15	16
English															
1. T1 phonological awareness	**.70**	**.46**	**.56**	**.33**	**.41**	**.73**	**.61**	**.76**	**.49**	**.32**	.23	.22	.11	**.53**	**.57**
2. T2 phonological awareness	-	**.40**	**.59**	**.39**	**.46**	**.58**	**.59**	**.68**	**.70**	**.45**	.18	.21	**.25**	**.39**	**.43**
3. T1 morphological awareness		-	**.73**	**.66**	**.57**	**.47**	**.48**	**.58**	**.35**	**.57**	**.27**	**.42**	**.35**	**.34**	.21
4. T2 morphological awareness			-	**.70**	**.66**	**.55**	**.63**	**.66**	**.48**	**.63**	**.35**	**.49**	**.51**	**.40**	**.38**
5. T1 vocabulary				-	**.73**	**.36**	**.45**	**.46**	**.28**	**.40**	.19	**.55**	**.49**	.18	.15
6. T2 vocabulary					-	**.54**	**.66**	**.47**	**.39**	**.43**	.19	**.61**	**.50**	**.40**	**.38**
7. T1 word reading						-	**.77**	**.64**	**.43**	**.41**	**.29**	**.26**	.17	**.58**	**.63**
8. T2 word reading							-	**.58**	**.56**	**.38**	.17	**.37**	**.32**	**.59**	**.70**
Spanish															
9. T1 phonological awareness								-	**.62**	**.64**	**.34**	**.52**	**.42**	**.57**	**.57**
10. T2 phonological awareness									-	**.47**	**.26**	**.44**	**.37**	**.37**	**.48**
11. T1 morphological awareness										-	**.64**	**.65**	**.67**	**.48**	**.34**
12. T2 morphological awareness											-	**.51**	**.48**	**.36**	**.28**
13. T1 vocabulary												-	**.75**	**.45**	**.45**
14. T2 vocabulary													-	**.45**	**.39**
15. T1 word reading														-	**.77**
16. T2 word reading															-

All correlations were controlled for age, gender, maternal education and working memory. Significant two-tailed partial correlations (controlling for age, gender, maternal education and working memory) are in boldface. Correlations between .25 and .32 are at the *p* < .05 level; correlations equal to and between .33 and .42 are at the *p* < .01 level; those greater than .42 are at the *p* < .001 level.

## Data Availability

The data supporting the findings of this study can be made available by the corresponding author upon reasonable request.
